# Sprengel’s Deformity With Congenital Scoliosis Successfully Treated With Combined Posterior Spinal Instrumented Fusion and Modified Woodward’s Procedure in a 14-Year-Old Patient With MURCS Association

**DOI:** 10.7759/cureus.15669

**Published:** 2021-06-15

**Authors:** Ozair Bin Majid, Zayed S Al-Zayed, Mohamed Alsehly, Shahd H Almonaie

**Affiliations:** 1 Department of Orthopaedic Surgery, King Faisal Specialist Hospital and Research Centre, Riyadh, SAU; 2 Department of Orthopaedic Surgery, Alfaisal University College of Medicine, Riyadh, SAU

**Keywords:** sprengels shoulder, scoliosis, murcs, woodwards procedure, deformity

## Abstract

Müllerian duct aplasia-renal agenesis-cervicothoracic somite dysplasia (MURCS) association is a rare syndrome. This unique condition consists of Müllerian duct aplasia, cervicothoracic somite dysplasia, and renal aplasia, and skeletal abnormalities manifesting in childhood. We report the case of a 14-year-old girl who presented to the orthopedic clinic with spinal deformity and Sprengel’s shoulder complicated by a background of MURCS association. The treatment modalities of scoliosis include posterior spinal fusion and the vertical expandable prosthetic titanium rib. On the other hand, Sprengel’s deformity is surgically managed by Woodward’s procedure. The management plan for our patient involved correcting scoliosis by the posterior spinal fusion procedure and performing Woodward’s procedure to correct Sprengel’s deformity simultaneously.

Simultaneous scoliosis correction with posterior spinal instrumented fusion and Sprengel's deformity correction with modified Woodward’s procedure is a promising surgical technique that can lead to favorable outcomes.

## Introduction

Müllerian duct aplasia-renal agenesis-cervicothoracic somite dysplasia (MURCS) association is a variant of the Mayer-Rokitansky-Küster-Hauser (MRKH) syndrome; it is a very rare condition causing renal agenesis, Müllerian duct agenesis, and cervical somite abnormalities. MURCS is known to be an inherited autosomal dominant condition that only affects females. It was first described in 1979 by Duncan et al. [1]. It can be associated with Sprengel's deformity and other spinal anomalies such as congenital scoliosis [2-4]. It can also be associated with Klippel Feil syndrome [5-7]. The most commonly used classification is the Cavendish classification; it divides the condition into very mild, mild, moderate, and severe forms. Woodward’s procedure, with or without clavicle osteotomy, has been one of the most commonly accepted procedures for Sprengel's deformity. Intraoperative neuromonitoring is very useful for monitoring the integrity of the brachial plexus during Woodward’s procedure by checking somatosensory evoked potentials (SSEPs) and motor evoked potentials (MEPs) [3-5]. Similarly, for congenital scoliosis, posterior spinal instrumented fusion is the mainstay of management to correct scoliosis and prevent further progression of the curve [8-10].

## Case presentation

**Figure 1:**
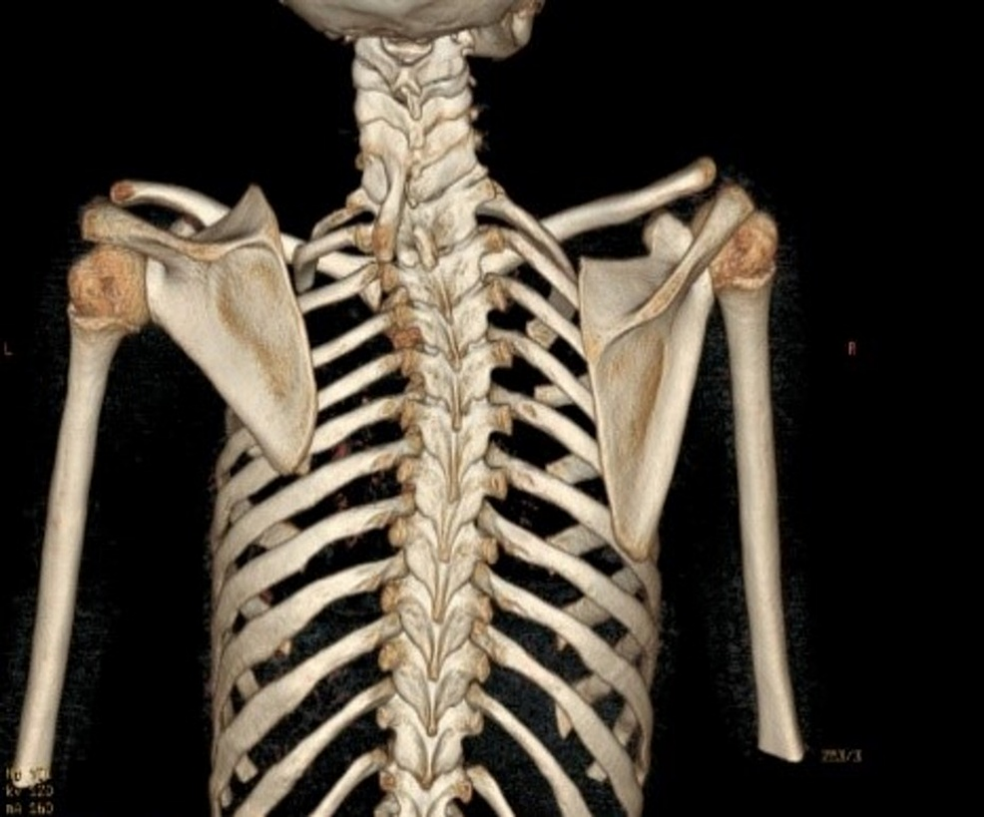
CT scan of the patient showing congenital scoliosis with unsegmented bars and elevated left scapula CT: computed tomography

**Figure 2:**
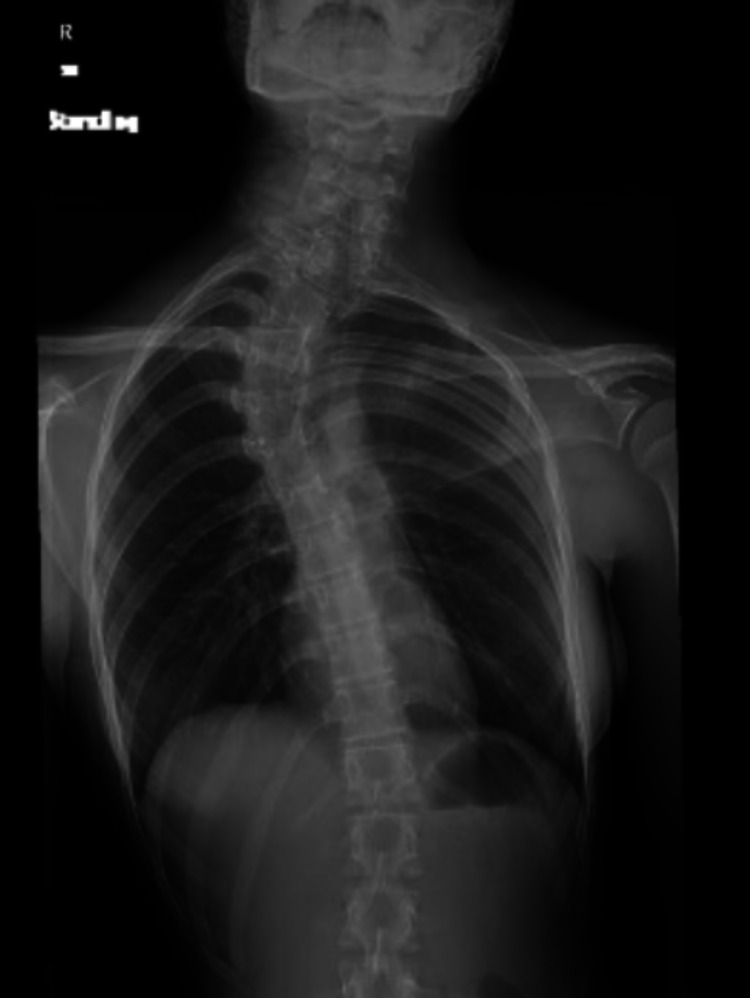
Plain AP radiograph of the patient showing congenital scoliosis and elevated left scapula AP: anteroposterior

A 14-year-old girl presented to the orthopedic clinic with spinal deformity. The girl was in her pre-menarche period and was under medical evaluation for primary amenorrhea. She had normal secondary sexual characteristics with Tanner stage 3. The patient’s family history and past medical and surgical history were all unremarkable except for a hernia repair performed at two years of age. The patient also complained of occasional back pain without any neurological deficits. On physical examination, she was found to have right upper thoracic scoliosis and her left shoulder was elevated. She had a large rib hump on the right side with waist asymmetry. Left shoulder abduction was limited to 100⁰ as compared to normal abduction on the right side. A plain radiograph and a CT scan were performed, which showed elevated left scapula with a small omovertebral bar connected to the C5 vertebral body (Figures 1, 2).

There was upper thoracic scoliosis of 56⁰ with partial fusion of C7 and T1 vertebral bodies. MRI scan of the spine was unremarkable for any intraspinal anomalies. Pelvic MRI showed agenesis of the uterus, cervix, and upper part of the vagina, along with left renal agenesis. The problem list included congenital scoliosis with 56⁰ upper thoracic curve, with left shoulder Sprengel’s deformity (Cavendish type 3) and left shoulder limited abduction. Posterior spinal instrumented fusion using bone graft combined with modified Woodward’s procedure was planned. During the operation, the patient was placed in a prone position under general anesthesia. The patient was draped in a manner so that the left shoulder girdle could be manipulated. Intraoperative neuromonitoring was employed to monitor SSEPs and MEPs for all the extremities. A midline incision was given from the upper cervical to upper lumbar vertebrae. We undermined the skin and subcutaneous tissues to the medial border of the left scapula. Following that, we subperiosteally dissected and retracted the muscles on both sides of spinous processes to the costotransverse process in the thoracic spine. On the left side, the rhomboids and trapezius muscles were freed from the chest wall muscles. An omovertebral bone was located attached from the cervical spine to the medial border of the scapula. Through extraperiosteal dissection, the omovertebral bone along with the fibrous tissue was excised. Levator scapulae muscle, which was found contracted, was also excised. Pedicle screws were inserted from T1 to T12 vertebrae through the freehand technique under fluoroscopy guidance. Soft tissue release including interspinous ligaments and spinous processes were resected in the thoracic spine. We used two cobalt chromium (CoCr) rigid rods and the translation technique to correct scoliosis. A moderate amount of correction was achieved. We attached a crosslink between the two rods. Afterward, we shifted our focus back to the scapula and released it further from the musculature. With the help of towel clips, the scapula was pulled downwards and rotated until the spinous processes of the two scapulae were on the same level. We then used fiber wire suture and passed it through the medial border of the scapula and tied it around the CoCr rod on the same side at around 45⁰ angle. To further strengthen our correction, we used Ethibond nonabsorbable sutures with the same technique as described for the fiber wire suture. Satisfactory descent of the scapula was achieved and no changes were recorded in SSEPs and MEPs with regard to the brachial plexus. We routinely use allograft bone chips for fusion; thus, in this case, we used the same after decortication. We used a cell saver throughout the procedure. The closure was done in layers using absorbable vicryl for fascia and subcutaneous tissue and monocryl absorbable for the skin.

**Figure 3:**
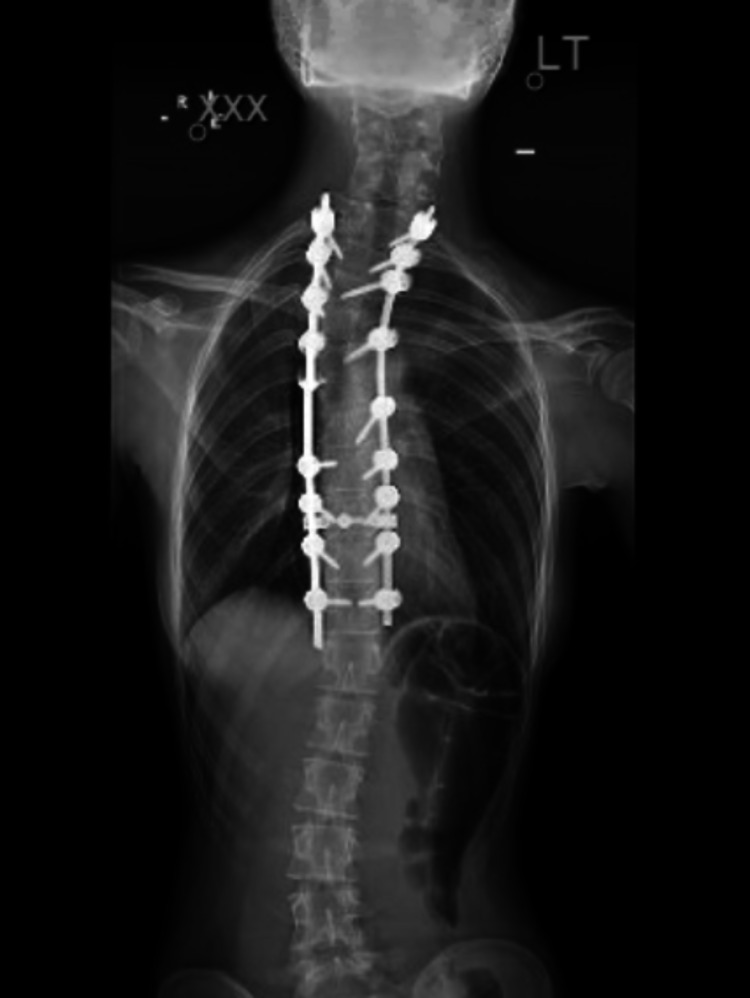
Plain AP radiograph of the patient showing posterior instrumentation for correction of scoliosis and correction of Sprengel's deformity* *The spines of both scapulae can be noticed at the same level AP: anteroposterior

Postoperative X-ray imaging showed good correction. The patient was actively moving all her extremities. A Velpeau bandage was applied and maintained for two weeks. Following that, a supervised shoulder rehabilitation program was started with active shoulder exercises. The patient was asked to continue the recommended exercises at home. After two years of follow-up, an X-ray was performed and showed successful correction of scoliosis and scapula and a clinically good shoulder abduction of 130⁰ (Figure 3).

## Discussion

MRKH syndrome, a rare disorder characterized by the absence of the uterus, is associated with a normal karyotype of 46,XX. MRKH is a syndromic umbrella with various other subtypes. MURCS association, the type 2 variant of MRKH, is a rare developmental disorder characterized by uterine aplasia, renal agenesis, vertebral anomalies, and short stature. The incidence of MRKH syndrome is unique and rare and as per the data in the medical literature, it has an occurrence rate of one out of 4,000-5,000 female newborns [2,4]. Sprengel's deformity is defined as a complex congenital anomaly of the shoulder girdle resulting in malposition of the scapula. Sprengel's deformity can result in functional deficits and cosmetic issues; therefore, a focused assessment and management of this congenital deformity warrant a careful approach. Our case report described the occurrence of Sprengel's deformity in association with MURCS association, necessitating a serious and critical management plan for a favorable outcome.

In this report, our main objective is to shed light on and emphasize the importance of further investigations with regard to the spinal deformities that can occur in patients suffering from MURCS association. We also wanted to highlight the procedure performed; in the standard Woodward’s procedure, the paraspinal musculature such as the trapezius and the rhomboids muscles are sutured to the spinous process more caudally in order to put the scapula in a downward position [11-13]. Ahmad, in his study, used a modified Woodward's procedure by anchoring the scapula to the lower dorsal vertebrae by stout suture through the superomedial scapula and achieved better shoulder abduction and correction of glenoid tilt [14]. Zhong et al. [15] studied 21 patients who underwent scoliosis correction combined with scapuloplasty for elevated shoulder (Cavendish score ranging from 2 to 3 points). In their proposed technique, after detaching the muscles covering the inferior medial angle of the scapula, the scapula was relocated caudally by suturing the muscle sheath to the same-side titanium rod using Ethibond sutures. The cosmetic appearance improved in all patients and good shoulder balance was achieved. Patients with a higher Cavendish score or congenital malformation of the scapula were excluded from their study. Our technique is the first of its kind to be used on a patient with MURCS association with congenital scoliosis and Sprengel's deformity. In the technique that we employed, after doing an extensive soft tissue detachment, we removed the omovertebral bar along with its fibrous band and released the contracted levator scapulae muscle. The released muscles were not reattached to the spinous processes and interspinous ligaments as they had been released to achieve the successful scoliosis correction. Instead, we placed the scapula distally with the help of fiber wire sutures and non-absorbable Ethibond sutures and tied them around the left CoCr rod at an angle to achieve correction. This strategy was adequate to bring the scapula inferiorly to its normal position and simultaneously correct the scoliosis deformity with posterior spinal instrumented fusion [3-5,7,8]. In light of the result obtained, we consider simultaneous scoliosis correction with posterior spinal instrumented fusion and Sprengel's deformity correction with modified Woodward’s procedure to be a promising technique that can achieve favorable outcomes. Further comprehensive prospective studies should be conducted to compare our technique’s clinical and radiologic outcomes. Additional information gathered through similar case studies will provide valuable contributions to the medical literature in the form of evidence-based comparisons.

## Conclusions

Through this report, we wanted to highlight the significance of conducting crucial investigations in patients suffering from MRKH syndrome and its variants. A multidisciplinary team approach is required to ensure successful treatment outcomes in the management of similar cases. Further study projects focusing on this topic should examine the handling of similar presentations with the proposed technique, i.e., concurrent scoliosis correction with posterior spinal instrumented fusion and Sprengel's deformity correction with modified Woodward’s procedure. Comparing and contrasting the future outcomes and documenting them for study baseline will contribute immensely to the literature on this topic.
